# Understanding Factors That Influence the Recruitment and Retention of Male Occupational Therapists in Aotearoa New Zealand

**DOI:** 10.1155/oti/8701982

**Published:** 2026-07-24

**Authors:** Simon Leadley, Patrick Broman, Darren Mills, Daniel Sutton

**Affiliations:** ^1^ Department of Occupational Therapy, School of Primary and Allied Health Care, Monash University, Frankston, Victoria, Australia, monash.ac.za; ^2^ Australian Centre for Student Equity and Success, Curtin University, Bentley, Western Australia, Australia, curtin.edu.au; ^3^ Te Kura Whakaora Ngangahau/School of Occupational Therapy, Otago Polytechnic, Hamilton, New Zealand; ^4^ School of Clinical Sciences, Auckland University of Technology, Auckland, New Zealand, aut.ac.nz

**Keywords:** men, occupational therapy, recruitment, retention

## Abstract

Low levels of recruitment and retention of men in the occupational therapy profession have been a long‐standing issue. This study examined the recruitment and retention of men in occupational therapy in Aotearoa New Zealand, with the aim to better understand what factors have been influencing this issue. The study employed an interpretive descriptive qualitative methodology. Focus groups were conducted with male occupational therapy students and practitioners. A thematic analysis approach was utilised. Strategies to ensure rigour and address cultural and ethical considerations were used throughout study. Two key themes emerged: *Identity* and *Diversity*. The first theme, *Identity*, highlighted limited public awareness about occupational therapy and its altruistic and pragmatic nature. It also drew attention to its perception as a predominantly female and white profession. These factors were identified as barriers to attracting and retaining men. Participants reflected on the transformative potential of occupational therapy, its hands‐on approach to problem‐solving, and the need for better promotion of the profession to appeal to men. The second theme, *Diversity*, addressed the lack of gender, ethnic, and cultural representation within occupational therapy. It also highlighted the advantages that greater diversity brings to practice. Participants emphasised that diverse perspectives are crucial for meeting the needs of Aotearoa New Zealand’s population. The wide range of roles and career opportunities within occupational therapy were also identified as factors that could encourage more men into the profession.

## 1. Introduction

The field of occupational therapy/whakaora ngangahau (the name in te reo Māori, the Indigenous people of Aotearoa New Zealand) plays a vital role in promoting participation and optimising performance in occupations (e.g., daily activities, education/work, recreational, social and cultural activities), to sustain people’s health, well‐being and quality of life [1]. Occupational therapy is dynamic, requiring a blend of theoretical and practice knowledge, empathy and a holistic approach to health care. In Aotearoa New Zealand, as in many countries, occupational therapy is a profession where the majority of therapists are female, with men significantly underrepresented [2]. While *vertical* segregation, the underrepresentation of women in leadership positions, is widely recognised and must be addressed, less attention has been paid to *horizontal* segregation, where fewer men enter and remain in health and social professions like occupational therapy [3, 4]. Gender representation in health professions is acknowledged as important for a number of reasons, particularly in terms of reflecting diverse views and meeting diverse population needs [2, 5, 6]. Despite this, the subjective experiences and understandings of men in occupational therapy, and the ways in which their numbers may be increased, is not well understood.

Amongst those working as occupational therapists, men are underrepresented both in Aotearoa New Zealand and internationally. Ratios of men to women are typically below 10% [2], although some countries, particularly in Asia do not follow this trend [7]. However, low ratios of men to women has been both a feature of the profession historically and is a contemporary reality, with these trends similar in other caring professions (i.e., nursing, dieticians and social work) [3, 5, 6, 8]. Table 1 below shows the latest Aotearoa New Zealand workforce data for proportions of men in a selected range of allied health professions [9–13]. The lack of gender diversity appears to be associated with disproportionately low recruitment and retention of men into the profession.

**Table 1:** Proportion of men in selected allied health professions in Aotearoa NZ (most recent workforce data).

	%
Occupational therapists	8.2%
Nurses	10%
Physiotherapists	30%
Social workers	15%
Dietitians	5%

Data provided for this study by the two occupational therapy training programmes in Aotearoa New Zealand (Otago Polytechnic and Auckland University of Technology) show the percentages of people graduating with a Bachelor of Occupational Therapy degree. For the Otago school, the percentage of males graduating over the 2012–2021 period was 7.1%, while males represented 13% of graduates at the Auckland school over 2012–2023. The differences in proportions of male graduates from occupational therapy schools may reflect the regional demographic composition of the areas where these schools are located. Drawing on the Occupational Therapy Board of New Zealand/Te Poari Whakaora Ngangahau O Aotearoa [11] publicly available data, there has been an increase in the percentage of male occupational therapists holding an annual practicing certificate (APC), rising from 7.6% in 2011–2012 period to 8.2% in 2023–2024. There is also a recent trend in occupational therapists holding an APC declaring their gender as ‘do not identify as male or female’ (i.e., up from 0% in 2021 to 8% in the 2024 period) [11].

Although research that clarifies the potential benefits of a more gender representative workforce is broadly lacking (see [2]), there is potential value from an increased proportion of male occupational therapists. For example, some studies have shown that males receiving occupational therapy services prefer to work with a male occupational therapist, or find they are able to develop positive therapeutic relationships more easily with male therapists [7, 8, 14]. Additionally, occupational therapy clinicians and students perceived value in terms of support and mentoring arising from a greater proportion of male occupational therapy clinical supervisors and educators [2, 7]. Furthermore, the recruitment of more men from ethnically and culturally diverse backgrounds [15, 16]; those from Indigenous backgrounds such as Māori in Aotearoa New Zealand [17, 18], and other minority groups (e.g., Pasifika) into the occupational therapy workforce has the potential to improve engagement and understanding of the different contexts experienced by people using occupational therapy services [19, 20].

Research to date on men in the occupational therapy profession, in particular related to the Aotearoa New Zealand context, is summarised by Leadley et al. [2]. It is noteworthy that the majority of research has been quantitative and survey‐based, with few published qualitative studies [21]. Work to date has outlined negatively gendered experiences for some men in occupational therapy, such as being expected to complete certain tasks based on their gender, or not expected to work with female clients. Other experiences reported in the literature relate to the impacts of role strain arising from being in a minority, including experiencing incredulous or negative responses from other people about working in occupational therapy [2, 7, 8, 14, 22]. This is possibly associated with public perceptions of occupational therapy as a female profession [14].

In terms of underrepresentation of men in the profession, it is important to point out that these issues are different to those related to labour market experiences more broadly, where women face systemic and longstanding gender inequity [23]. Therefore, it is more appropriate to consider this issue a matter of diversity, as men are not necessarily facing issues of inequity or injustice by being in the minority. For example, men often gain promotions in the profession (e.g., ‘glass escalator’ effect) [8]. Addressing the issue of horizontal segregation and raising gender diversity is important, however, the proportion of men in occupational therapy and other health professions (e.g., nursing) has not differed much over the last 20–30 years [8]. Systemic level changes are required, and this requires an understanding of factors influencing male recruitment and retention into occupational therapy.

Previous studies exploring drivers of poor male recruitment into and retention in occupational therapy have suggested causes include ad hoc recruitment strategies for men, experiences of discrimination (as students or clinicians), concerns expressed by men when considering the profession such as low salaries or limited opportunities for career progression, and the potentially lower social status of occupational therapy compared with other professions [2, 7, 15, 22, 24]. Regarding retention rates, it is important to note that a study of Australian female (*n* = 130) and male (*n* = 65) occupational therapists revealed no difference in job satisfaction rates based on gender, and indeed found male participants to be more optimistic about job opportunities than their female counterparts [25].

A recent Aotearoa New Zealand‐based survey (*n* = 22) of male occupational therapy students at the Otago School of Occupational Therapy [2] revealed that these students saw themselves being suited to the role and generally had a positive outlook on entering the profession. While the majority of Aotearoa New Zealand occupational therapy male students in the study felt supported in the teaching environment, some felt ‘ostracised and isolated’ due to being in the minority amongst their peers [2]. This survey did not however allow for detailed description of the experiences of male occupational students, and as a point‐of‐time study of then‐current students, could not consider the experience of clinicians who had entered the profession earlier, or their experiences once entering the workforce.

Research into the recruitment and retention of men into various female majority health professions (e.g., nursing, dietetics etc.) have explored various solutions to the problem. These have included policy that promotes these health professions to men (e.g., altruistic nature of the roles, career, salary, flexibility), addressing persisting gender stereotyping, and providing mentoring and support [5, 6, 26, 27]. Little is known, however, about the likelihood of success for these various initiatives in occupational therapy, given the limited picture surrounding the individual‐level causes and drivers of low male recruitment.

Given the currently limited picture regarding male retention and recruitment, this study is intended to provide an in‐depth examination of the current views of male students at the occupational therapy schools, as well as a sample of practicing male occupational therapists. We sought to gain a greater understanding about recruitment and retention factors, particularly from an Aotearoa New Zealand perspective and context, and to identify possible solutions to recruiting and retaining men into the profession. This study’s findings may support approaches to the issue taken by occupational therapy schools, and other stakeholders in the profession (e.g., professional associations or regulatory bodies), as well as employers. The key research question posed was: *What are the tane* ^1^ */male perspectives on how to best recruit and retain men into the profession of whakaora ngangahau/occupational therapy?*

## 2. Study Design

The theoretical perspective underpinning the study design was interpretivism, and more specifically an *interpretive descriptive qualitative methodology* was adopted. As described by Thorne [28], interpretive descriptive qualitative methodology focuses on generating practical and clinically relevant insights rather than developing formal theory (such as grounded theory) or identifying a universal essence of experience (phenomenology). Interpretive descriptive qualitative methodology is particularly useful when the aim is to inform practice, policy or professional understanding [28, 29]. It was chosen here as the study aimed to generate practical insights into factors influencing the recruitment/retention of local male occupational therapists: a real‐world practice context.

Interpretive descriptive qualitative methodology involves the use of flexible methods to identify patterns, meanings and contextual influences across participant experiences [28]. The primary method adopted in this study was qualitative focus group data collection and analysis. Focus groups are intended to elicit and examine participants’ perspectives in depth, rather than to foster debate or arrive at a consensus [30]. Focus groups allow participants to discuss, challenge and build on each other’s perspectives, helping researchers understand how views are formed within social and cultural contexts. The choice of focus groups over other qualitative methods, such as individual interviews, was made to facilitate collective discussion and the sharing of ideas. Focus groups enabled participants to generate and explore perspectives in a group context, allowing for richer interaction and exchange compared with one‐on‐one interviews. Consistent with an interpretivist approach, where the aim to identify “culturally derived and historically situated interpretations of the social life‐world” (Crotty [31], p. 67), the focus group setting helped reveal how meanings around gender, professional identity, recruitment and retention were socially constructed through interaction.

## 3. Researcher Positionality

All authors are white, culturally and socio‐economically privileged males. Three (S.L., D.N., D.S.) work in academic roles as occupational therapy lecturers and one (P.B.) has a research and teaching background and works in education. The authors believe that diversity is important in the occupational therapy workforce, including in life experience, culture, sexuality and gender. This study was motivated by the desire to support a range of males into the profession to reflect the diversity of men accessing occupational therapy services. Key assumptions held by the researchers included the following: marketing directly to men by male OTs would help recruitment, male support and role modelling for male OT students and clinicians would support retention, and drivers for both recruitment and retention of men would include helping others, employment opportunities and OT role diversity.

## 4. Participants/Recruitment

A purposive sampling approach was adopted for this study, whereby the two local occupational therapy programmes (for students) and the Occupational Therapy Board of New Zealand (clinicians) supported recruitment for the study via group distribution emails to all students enrolled in their programme or registered as occupational therapists. Inclusion criteria required participants to identify as male and be either practising occupational therapists or occupational therapy students in Aotearoa New Zealand. Individuals without current or recent experience within the profession or training pathway were excluded. Per Allen [30] focus groups will ideally comprise a purposeful sample of 6–8 participants with a background and experience relevant to the topic of interest. Recruitment emails provided a participant information sheet about the study and invited those interested in participating in a focus group to contact a non‐occupational therapy, non‐educator member of the research team via email if they were interested in participating. This researcher then contacted them with further information and to arrange focus groups. Key information about each participant was gathered voluntarily via survey questionnaire distributed with the written informed consent form completed prior to data collection. Nineteen participants (seven students and 12 clinicians) participated in four focus groups. This sample size was considered appropriate for an interpretive descriptive study in New Zealand, where there are fewer than 4000 registered occupational therapists [11]. It enabled exploration of perspectives across different career stages and practice contexts, with recurring patterns relating to recruitment and retention experiences consistently identified across groups. See Table 2 for participant characteristics.

**Table 2:** Participant characteristics.

Students	*N*	%
Ethnicity		
New Zealand European	5	71%
Other	2	29%
Provider		
AUT	3	43%
Otago Polytechnic (Dunedin)	1	14%
Otago Polytechnic (Hamilton)	3	43%
Year of study		
1st year	3	43%
2nd year	1	14%
3rd year	2	29%
4th+ years	1	14%
Total	7	100%
Clinicians	N	%
Ethnicity		
New Zealand European	8	67%
Māori	1	8%
Other	1	8%
Did not answer	2	17%
Years in practice		
0–4 years	1	8%
5–14 years	2	17%
15+ years	7	58%
Did not answer	2	17%
Field working in		
Physical health	3	25%
Mental health	7	58%
Did not answer	2	17%
Primary client base		
Children	3	25%
Adults	7	58%
Did not answer	2	17%
Total	12	100%

Written informed consent was obtained from all participants. Confidentiality was maintained in the focus groups, by ensuring the conversations remained closed to only participants in each focus group and the researcher conducting the groups (P.B.). Members of the research team may have been known to potential participants as fellow New Zealand registered occupational therapists (clinicians) or as teaching staff in their programme (students), so a non‐occupational therapy researcher (P.B.) was engaged to facilitate data collection. Except for this researcher, participant identities were blinded to other members of the research team.

## 5. Data Collection

Four focus groups were conducted, each led by a single researcher who was not previously familiar with the participants. Following Allen [30], groups of between five and eight participants were sought. Two of the focus groups were conducted with students and two with clinicians (during 2022). The first student group (*n* = 3) was facilitated face‐to‐face at the campus of Otago Polytechnic’s Hamilton programme, and the second was held online via Zoom with four students from the two other programmes. The clinician groups (*n* = 7 and 5) were both conducted online via Zoom.

All focus groups were around 1 h in length. A semi‐structured framework of open‐ended questions was devised by the researchers following a preliminary review of the literature. Reflecting the qualitative interpretive descriptive approach, where questions are typically flexible and open‐ended, the instrument was not field tested but refined throughout the process. Because of the length, the final interview guides are included as Appendix 1. End‐to‐end encryption was enabled for Zoom interviews and recordings were available only via password access. The in‐person interview was recorded via voice recorder. A voucher to the value of NZD$30.00 was offered to participants in recognition of their time. To maximise trustworthiness, constructive checking was undertaken during interviews, and all participants were offered a copy of the transcript to review.

## 6. Data Analysis

Focus group recordings were transcribed verbatim, with each participant assigned a pseudonym to maintain confidentiality. Resulting data were scrutinized using a thematic analysis approach, as described by Braun and Clarke [32] and Nowell et al. [33]. Thematic analysis provides a means for identifying themes within and interpreting qualitative data, without focusing on the development of full‐scale theory. It is especially suited for exploratory research [34]. An inductive six‐step process, as outlined by Braun and Clarke [32], was undertaken, with a qualitative interpretivist emphasis on understanding male participants’ own subjective understandings, beliefs, experiences and positionality. The use of an inductive analysis strategy in this study aligns well with the interpretivist epistemology underpinning the methodology and epistemological stance adopted by the researchers. It allows themes to emerge directly from the data through an iterative process of engaging with transcripts, coding and developing thematic categories. Furthermore, it supports a reflexive approach, recognising that researchers are actively involved in the interpretation of the data [28, 35]. Interpretive descriptive methodology aligns with a reflexive thematic analysis approach, as both extend beyond purely descriptive analysis to interpret meaning within the data. Both approaches value researcher subjectivity and reflexivity, and emphasise the generation of practical, meaningful knowledge rather than theoretical abstraction. They also share an inductive and iterative approach to data analysis, whereby insights are developed progressively through ongoing engagement with the data.

Two of the researchers were occupational therapists and educators (S.L., D.M.), while the third was a researcher with a teaching background (P.B.). Their working relationship developed through a shared passion for the topic, a commitment to ongoing research in this area, and a dedication to equitable decision‐making processes, supported an equitable power dynamic. All three researchers carried out each of the steps, being (1) detailed familiarization with the data (transcripts), (2) generating initial codes, (3) searching for themes, (4) reviewing themes, (5) defining and naming themes and (6) producing the report. See Table 3 for further description and examples of the thematic analysis process. All three researchers independently read and familiarised themselves with the transcripts and conducted initial coding. They then met to compare and refine codes as needed, employing a form of intercoder comparison. Through deliberation and consensus‐based decision‐making, they resolved discrepancies and reached agreement on the final coding scheme. Themes were then identified and agreed collectively at a subsequent meeting before all researchers re‐read the transcripts and individually reviewed themes. Further discussions ensued before final themes were defined and agreed. An audit trail was maintained throughout the study, documenting each stage of the research process, including study protocols, ethical procedures, data collection and analysis and report writing. Records were kept at each stage of the study documenting key decisions made (e.g., email records, memos and records of each stage of the study such as analysis and saved in specific study folders). All focus group participants were given the opportunity to review the transcript of the focus group in which they participated (i.e., member checking). No participants across the groups requested any changes. The research team remained reflexive throughout the study by engaging in discussions related to their assumptions about the topic, and being open to diverse interpretations of the data. However, the researchers were aware of the results of previous research on the topic conducted by two of the researchers (S.L. & D.M.). That revealed male OT student participants viewed key recruitment and retention strategies as targeted marketing to men, highlighting the breadth of roles in the profession, a desire to help others, job security and mentoring of male OT students. Additionally, relevant literature was reviewed and drawn on in the interpretation of the emerging findings (a form of theoretical triangulation). This literature provided theory and varied perspectives (e.g., a feminist critical perspective), which helped to incorporate diverse viewpoints into our reflexive process. While the reflexive process was informed by the researchers’ own experiences as white, male academics and occupational therapists, this process could have been strengthened by including occupational therapy researchers from female and other ethnic/cultural backgrounds in the team, thereby broadening the range of perspectives informing the interpretation of the data. The level of data gathered from all focus groups was considered sufficient to answer the research questions, as the sample included occupational therapy students from both OT programmes and clinicians from across the country, enabling a range of student and clinical perspectives from diverse backgrounds. However, there were pragmatic limitations to the number of focus groups, including financial constraints and disruptions during the COVID‐19 pandemic. More focus groups may have increased the number of participants in the study and allowed for the inclusion of a broader range of perspectives, such as greater ethnic and cultural diversity amongst students and clinicians. All authors then contributed to the final manuscript.

**Table 3:** Thematic analysis process.

Steps of thematic analysis	Process applied and examples from study
Detailed familiarization with the data (transcripts)	All three researchers immersed themselves in data, via reading and re‐reading of transcripts. Keeping an open mind and an awareness of assumptions related to the research question, researchers documented and shared their initial ideas.
Generating initial codes	Initial coding was then conducted independently by each researcher. The team then met to discuss codes using visual mapping methods to group these into early themes. For example; ‘Difficult to define OT’, ‘Nobody’s heard of OT’ ‘Do not have a strong recognisable identity’–grouped under ‘*OT lacking identity’*. Final codes were agreed through ongoing discussion and reflexivity related to different perspectives. A process of reaching consensus through reflexive discussion enhanced analytical rigour and consistency in interpretation.
Searching for themes	An initial deductive framework (Push, Pull and Recruitment and Retention) guided early grouping of codes. This was followed by an inductive process, allowing new themes to emerge from the data beyond the predefined framework. For example, *Recruitment and Retention – Identity in Occupational Therapy–OT lacks identity:* sub‐themes: *Diversity of profession makes it hard to define identity, OT difficult to define, professional identity not well understood.*
Reviewing themes	Themes and sub‐themes were refined through an iterative process, involving checking transcripts to ensure multiple sources of data verified the themes, and a reflexive process where researchers considered how their own professional backgrounds and assumptions shaped interpretations. Example of initial key theme: *Identity in Occupational Therapy*; sub‐theme–*Occupational Therapy lacks an identity. Example verifying data: “You do not hear OT…being advertised…it’s not very widely known what we actually do” (Clinician); “there’s not really a massive understanding of it…not a lot of advocacy…its quite hard to…understand” (Clinician); “I’d say it’s the same old story of where OT is…still not that well known” (Student); “huge barrier is the [lack of] awareness of the profession” (Student);*
Defining and naming themes	Finalising themes and sub‐themes, with agreement by consensus. For example, Key theme: *Identity as Occupational Therapists,* with sub‐theme *Professional Identity: Not Well Understood*. Examples of supporting data extracts: *“[We] do not have a strong recognisable identity. I think that’s … a big concern.” (Student 3); “It’s not very widely known what we actually do.” (Clinician 7)*
Producing the report	Report co‐written by researchers. Ensuring clearly documented themes and subthemes, explained sufficiently and supported by a range of quotes from both clinicians and student participants across all four focus groups.

## 7. Rigour and Trustworthiness

Rigour or trustworthiness in the outcomes of a study is paramount if they are to be trusted and useful to professional practice [36]. To help achieve trustworthiness in this study, we used a transparent research process, challenged our assumptions and explored alternative explanations for findings, and used regular quotations from participants in the write‐up. We also maintained a reflexive approach as researchers, accessed supervision and peer review, sought ethical and cultural advice and maintained an auditable trail of data gathering and decision‐making.

## 8. Ethical and Cultural Considerations

Cultural consultation was undertaken through the Māori research advisory unit, Kaitohutohu, at Otago Polytechnic. Ethics approval for this project was obtained for Otago Polytechnic students and for clinicians, from the Otago Polytechnic Research Ethics Committee: reference number 921, dated 13 September 2021. Separate approval related to AUT students was obtained from the Auckland University of Technology Ethics Committee (AUTEC), application 22/249, 11 October 2022.

## 9. Results

Two key themes emerged from the data analysis: (1) *Identity as Occupational Therapists*, with sub‐themes of: *Professional Identity: Not Well Understood*; *Wanting to Make a Difference*; and *Practical, Hands on Problem-Solvers*; and (2) *Diversity in Occupational Therapy,* with sub‐themes of: *A Lack of Diversity*; *Benefits of Diversity in the Profession*; and *Diversity of Roles in the Profession*.

### 9.1. Identity as Occupational Therapists

A key concept or theme that emerged from the focus groups was that of identity. In relation to recruitment and retention of men into the profession, male occupational therapy students and clinicians spoke of the need to more clearly explain and promote the profession to the public, to broaden its appeal to men by promoting a more diverse identity, and one that better articulated the altruistic aims and pragmatic skills of the profession. The three sub‐themes that emerged in relation to this theme will now be discussed.

#### 9.1.1. Professional Identity: Not Well Understood

A primary part of the discussion about identity in the profession was that the term ‘occupational therapy’ was difficult to define personally, was not well understood by the public and tended to not be very visible in the public eye (e.g., in the media, television/movies) or well promoted.

*[We] don’t have a strong recognisable identity. I think that’s … a big concern.* (Student 3)

*I’d say it’s the same old story of where OT is … still not that well known.* (Student 4)

*Even OTs talk about not quite knowing what OTs do.* (Student 1)

*It’s not very widely known what we actually do.* (Clinician 7)

*It’s not out there in the public eye … in terms of visibility… its … harder to define … to [say] … exactly what an OT does.* (Clinician 11)

From their perspective, this was problematic both for the profession in promoting the valuable contribution occupational therapy makes in people’s lives, but also to attracting a greater number and a more diverse range of people into the profession, including more men. Links between the lack of an external (mainstream) professional identity and their own ability to define the profession highlighted difficulties promoting the profession and impacted recruitment.

*We seem to cringe a little bit when people ask what is an OT … we should be very clear in terms of what an occupational therapist is … and consistent in terms of that definition … that’s our way of actually being able to, really promote the profession and get more people in, particularly men … I see that as being a real weakness for OT … that’s not ideal if you’re trying to explain to the general public what OT is.* (Clinician 4)

*The core of OT is probably actually quite appealing to lots of guys … importance of being caring, of looking after other people … it’s actually a real big value for … lots of guys … looking after others in practical ways … so that actually fits quite closely to the core of OT … actually supporting and helping people in quite practical, tangible ways, it is a bit funny, that side of OT hasn’t been, publicised I guess.* (Student 1)

From the participants’ perspectives, occupational therapy was associated with women of European ethnicity. They saw this as something that men could not easily relate to, and likely a contributing factor in ongoing low recruitment.

*[The profession is] mainly sort of middle aged white females.* (Student 2)

*I had doubts as well … particularly in those first few years around whether in fact OT was going to be right for me. Being a bloke surrounded by women, getting into it, an occupation that traditionally has been, well and still is, a very female dominated profession to get into.* (Clinician 4)

#### 9.1.2. Wanting to Make a Difference

While the historic gender base in the profession was an identifying feature that was seen as a barrier to recruiting and retaining men, contrastingly the male participants understood the value of the profession being transformative in the lives of persons they worked with. There was a strong consensus that an altruistic desire to make positive changes in people’s lives was one primary reason that many of them entered the profession.

*The work you do can be kind of life altering, so every day there’s an opportunity to make a difference you know and it’s not just bloody medication, here’s your meds you know like get rid of the symptoms—you know we can be really creative … And that’s what kept me in the game.* (Clinician 6)

*The best of myself [is] making a difference in someone else’s life.* (Student 3)

*So, what is the pull that gets you over that hurdle—it’s the difference you can make.* (Student 2)

#### 9.1.3. Practical, Hands‐on Problem‐Solvers

Another important part of the field of occupational therapy that participants believed could help attract men was the practical, doing and problem‐solving skill base that is involved. They believed this aspect of the profession would appeal to some men, if it was better articulated in marketing.

*[It’s a] hands on job and you can make a difference … [and it involves] a whole lot of practical skills.* (Clinician 7)

*[We need a] marketing approach that would appeal to males that has [a] more problem solving … very practical approach … we are problem solvers, we have a practical approach and assist with independence.* (Clinician 4)

*OT, I think it’s the hands-on thing that gives us the edge … that’s probably the biggest appeal … because OT is about doing … That actually would appeal to a lot of … guys that kind of problem solving, practical stuff if they actually knew that was there.* (Student 3)

While agreeing that these traits are likely helpful in recruiting men into the profession, at least one participant in the focus groups did not have the same affinity with these practical skill‐based characteristics, instead identifying with a more caring, sensitive nature.

*I don’t class myself as a typical sort of bloke at all, myself. You know I’m gay [and] I am not particularly practical with my hands. I’m the kind of softy, sensitive, caring guy. But you’re right, that actually would appeal to a lot of guys—that kind of problem-solving, practical stuff, if they actually knew that was there.* (Student 3)

### 9.2. Diversity

A second key concept or theme that emerged from the focus groups was diversity in the profession. In relation to recruitment and retention of men, the male occupational therapy students and clinicians spoke of the lack of both gender and ethnic diversity in the profession. They were clear in their belief that the profession would benefit from increasing practitioner diversity. An additional feature of this diversity theme was the scope for varied roles in the profession, that was seen as a key recruitment factor, and that also helped retain men in the profession.

#### 9.2.1. A Lack of Diversity

The perception of participants in this study that occupational therapy lacked gender diversity was seen as problematic, as it did not reflect society.

*Most practicing OTs are women … not being able to imagine yourself into that profession because you don’t even know what OTs do and then the other one is all the OTs you see are women.* (Student 1)

*I trained … started training in 1999. And there were 40, full time students in the year at that time. And three were men. (Clinician 9)*

#### 9.2.2. Benefits of Diversity in the Profession

There was a strong sense amongst the participants that increasing diversity in the profession by recruiting more men would help it better meet the diverse needs of the public. For example, participants drew on personal experiences where their male gender identity helped to build an effective relationship and thus a positive outcome in the occupational therapy process with male clients.

*Diversity … you know, naturally there are those situations where having someone of a similar gender or a preferred gender is going to make somebody [feel] more comfortable.* (Clinician 9)

*Some clients do speak better to males, some speak better to the females, it’s just, having that kind of diverse range … it’s definitely beneficial.* (Clinician 12)

*Working quite closely with some male clients, yeah they respond really, really well just to have another male OT present or just another male in general just to be able to sit down [together].* (Student 4)

Ethnic and cultural identity was also identified as an important feature when working with clients, where one Indigenous Māori participant discussed the benefits of being a tane (male) Māori kaiwhakaora ngangahau (occupational therapist) when working with Māori.

*Māori working with a Māori … [an OT] friend of mine I was talking about … he worked in the prison, he is Māori, so for him he goes in there and he just says ‘I’m working with my people’, and for him he loves that, he loves the idea that he’s making a difference with his own people*. (Student 3)

#### 9.2.3. Diversity of Roles in the Profession

One key reason why men in this study entered and stayed in the profession was the diverse range of roles that exist for occupational therapists, as well as opportunities to work in different contexts, including travelling and working internationally. The participants saw these features of the profession as being potentially very positive factors in terms of recruitment and retention of men into the profession.

*I think one of the good things about OT is just the diversity you can get into and certainly when I’m talking with people about OT it’s you know that catches people’s interest, just the range of ages, and mental health, physical health, education, you name it there’s OTs.* (Clinician 10)

*I think a huge part for me is the diversity and flexibility … you can pretty much work in lots of different areas, places around the world, pretty much lots of different environments—schools, businesses, hospitals.* (Clinician 8)

*It’s such a specialised role, but it’s also so broad so I think … everyone’s going to find something that they enjoy in it.* (Student 7)

## 10. Discussion

This study examined the views of occupational therapy students and clinicians in Aotearoa New Zealand related to factors that affect the recruitment and retention of men in the profession. Two key themes emerged, that of identity and diversity.

For men in this study, the concept of identity was linked primarily to a belief that society lacks understanding of occupational therapy as a unique science and health care vocation. This lack of understanding is likely to have a negative impact on attracting men, including those from diverse ethnic backgrounds, into the profession. The difficulty in explaining occupational therapy to the public and consequently the lack of clarity about, valuing of and promotion of the profession publicly has been identified as an issue in the recruitment of men in other studies [14, 20, 25]. Participants emphasised this point, and the need for the profession to promote occupational therapy and its benefits in a straightforward way to the public. There was a broad consensus that this would be beneficial in strengthening the profession’s identity and aid recruitment of men into occupational therapy.

A second element of identity related to the image of occupational therapists as health workers with pragmatic, ‘hands‐on’ problem‐solving skills. This was seen as a distinctive trait to the profession and one that was viewed as being attractive to many men, which could serve to drive positive recruitment. While Bäckström et al. [21] did identify the problem‐solving nature of the profession as an important factor in recruiting a diverse workforce, this study goes further to suggest that the pragmatic, ‘on the ground’ and practical nature of occupational therapy could also be important aspects to consider in the recruitment of a diverse range of men. Despite this finding, a narrow focus on such traits in recruitment risks reinforcing gender stereotypes and should be balanced with equal emphasis on relational, emotional and empathetic aspects of the profession. This would align with recent moves to broaden perspectives and enactments of caring masculinities [37, 38].

The third aspect of the identity theme related to the belief shared by those in this study that they could make an important and lasting influence in lives of the people they served as occupational therapists. This was a key reason for many of them joining and remaining in the profession, and many thought that if this was explained clearly, it would be an important pull factor in the recruitment for other men entering and remaining in occupational therapy. Other studies have pointed out that altruistic aims are motivators for entering the profession across all genders [14] and that this is important for those from minority groups, including men [7, 15].

However, the strong emphasis on having real and lasting positive influence in people’s lives in the present study contrasts with other authors who have suggested that men are less likely to become occupational therapists due to a focus on career factors (i.e., material and career advancement) rather than ‘calling’ aspirations, such as altruistic and moral reasons [20]. It may be that men already entering or working in the profession, such as those in this study, may be more motivated by such aspirations [8]. With this in mind, asking men outside of the profession why they would, or would not, consider a career in occupational therapy may also be fruitful in uncovering perceptions and barriers to recruitment. While Bäckström et al. [21] found that both men and women are motivated by altruistic aims, the emphasis that the male participants put on this as a pull factor suggests that it needs to be fore fronted in the recruitment of men into occupational therapy.

The theme of diversity included the perceived need for a more diverse profession as an important pull factor for male recruitment (e.g., having positive images of men in the profession) and retention (e.g., more male occupational therapy university lecturers, fieldwork and clinical supervisors). This may help to explain why levels of men in the profession continue to be low. Equally, diversity also meant benefits for the profession (e.g., a broad range of perspectives on occupational science and therapy) and for the people occupational therapists work with. While one study found gender was not a factor for men in choosing to enter the profession [7], most studies on the topic have identified the issue of the profession being majority female as being problematic for the recruitment of men [2, 8, 20–22]. This body of work highlights the need for systemic responses to horizontal segregation and to the deeply gendered construction of caring professions, such as occupational therapy, within society [38].

Increasing diversity in the profession to include a greater proportion of men (as well as those of different ethnic/cultural backgrounds), has been highlighted by other researchers as important in supporting the aims of occupational therapy within our diverse communities served, by helping to bring an enriching range of perspectives [15, 16, 20, 21]. As others have pointed out, a process of decolonizing occupational science and therapy, in Aotearoa New Zealand and internationally, is urgently required to better represent Indigenous, and diverse world views and perspectives [39, 40]. This would likely have a positive impact on the recruitment of a diverse workforce, including tane (male) Māori in Aotearoa New Zealand.

A final, but different meaning relating to the theme of diversity for the men in this study was the range of roles and opportunities in the profession as a recruitment and retention pull factor (i.e., differing fields of practice/roles, flexibility of practice, opportunities for working overseas). A study by Colaianni et al. [15] did reveal that some occupational therapy students of minority backgrounds (including men) saw varied populations and fields of practice as a factor in entering the profession. However, the male students and clinicians’ emphasis on this as an important pull factor was a unique finding of the present study.

Finally, functional and pragmatic push factors in recruitment and retention of men in the profession have been identified by other authors [2, 8, 15, 20, 22]. These factors include concerns with low remuneration, lack of career progression, feeling stigmatised or vulnerable as men in a female majority profession, low job security or satisfaction and role strain/ambiguity/conflict. Interestingly, these factors were not highlighted by the men in the present study. This may suggest that efforts to attract males are more likely to benefit from an emphasis on positive ‘pull’ factors, as opposed to focusing solely on addressing or minimising perceptions of negative factors.

## 11. Strengths and Limitations

The study findings add to the limited evidence related to factors influencing recruitment and retention of male occupational therapists. While focused on the Aotearoa NZ context, the alignment of several themes with studies from other countries suggests that at least some of the push‐pull factors may be relevant to contexts outside of Aotearoa. With the participants being mostly of European descent, the transferability of findings may be strongest in Western contexts where similar cultural and gender norms exist. The study also has some limitations including the relatively small number of participants, which meant that they were less likely to be representative of the wider male occupational therapy population in Aotearoa New Zealand. The sample was also limited in terms of ethnic diversity and cultural perspectives, with most students and clinicians stating their ethnicity as ‘New Zealand European’, or ‘Other’, and with only one Māori participant involved. Furthermore, while this study aimed to only include male participants, the views of females or those of other diverse gender identities, people with differing abilities or from those with a lived experience of occupational therapy services would have provided a wider range of perspectives on the topic. Additionally, as authors, we acknowledge our position as white, Pākehā men, of privileged positions, and that a more diverse authorship would have added breadth and depth in the interpretation of findings.

## 12. Conclusion

Similarly to other helping professions, men remain underrepresented in whakaora ngangahau/occupational therapy in Aotearoa New Zealand. Given that increased practitioner diversity and representation in the profession can improve its capacity to positively impact the health and wellbeing of those using services, it is important to understand the factors influencing the recruitment and retention of men to the profession. Adopting an interpretive descriptive qualitative methodology to discuss these factors with male students and practitioners in Aotearoa New Zealand via focus group discussion revealed two key themes: Identity as Occupational Therapists, and Diversity, along with several important subthemes. A complex picture of push and pull recruitment and retention factors influencing the underrepresentation of men in the profession emerged, that in part echoes findings from previous studies on the topic. But also provides several innovative initiatives that may help address the issue such as promoting the practical, problem‐solving and altruistic aspects of the profession to men of diverse backgrounds.

## Author Contributions

All authors made substantial contributions to the design and completion of the research. All authors were involved in drafting the manuscript and revising it critically for intellectual content.

## Funding

Funding was provided for this project by the School of Occupational Therapy, Otago Polytechnic. The funder had no involvement in the study design, data analysis or the decision to publish. Open access publishing facilitated by Monash University, as part of the Wiley ‐ Monash University agreement via the Council of Australasian University Librarians.

## Conflicts of Interest

The authors declare no conflicts of interest.

## Endnotes

^1^Tane means men in the Māori language.

## Data Availability

The data that support the findings of this study are available on request from the corresponding author. The data are not publicly available due to privacy or ethical restrictions.

## Appendix 1

**TABLE A1:** Semi‐Structured Interview Questions.

Number	Question
1	How were you initially drawn to training/working in occupational therapy? What were some of the pull factors?
2	How did being male affect or influence your decision?
3	As males, how have you experienced your training/working as an occupational therapist?
4	Are there any supports you have found helpful? Are there any other supports/ways you could be supported by staff at the school? [Students]
5	Is there enough diversity in occupational therapy? In what ways?
6	Do you think it’s important that OT as a profession represents or matches the population it serves? Does it?
7	Are there ‘enough’ men in the placement or training settings you have been in?
8	We’re interested in how gender relates to roles and responsibilities at work. Do you think there are certain skills that male or female OT might find it easier to provide for clients?
9	Has being male has influenced your career progression or trajectory. Can you talk about that? [Clinicians]
10	Could more males be helped to training/working in OT? How?
11	Thinking about retention for yourselves or for males, or for OT more generally, some people might say retention of occupational therapists in study/the profession is a challenge. Can you talk about that?
